# Lymphadenectomy Benefits Small Cell Carcinoma of Ovary: A Population-Based Analysis

**DOI:** 10.3390/curroncol29100617

**Published:** 2022-10-16

**Authors:** Jing Wang, Yan Ning, Yan Du, Yu Kang

**Affiliations:** Obstetrics and Gynecology Hospital, Fudan University, No.419, Fangxie Road, Shanghai 200011, China

**Keywords:** small cell carcinoma of the ovary, lymphadenectomy, SEER, propensity-score matching analysis, overall survival

## Abstract

Small cell carcinoma of the ovary (SCCO) is a rare type of ovarian cancer with high aggressiveness. The optimal treatment modality remains elusive. This study aims to comprehensively investigate the survival impact of clinical characteristics and treatments including lymphadenectomy in SCCO. A retrospective cohort study was performed and included patients from the Surveillance, Epidemiology, and End Results (SEER) database. Data collected included demographics, therapeutic details, and pathologic characteristics. Propensity-score matching analysis (PSM) was carried out to balance baseline variables between SCCO and non-SCCO. Cox regression, Kaplan–Meier, and stratified analyses were conducted before and after PSM. After filtering, 80 records on SCCO and 39,662 records on non-SSCO were obtained. Patients with SCCO were more prone to present unilateral tumor (57.6% and 85.0%, *p* < 0.001), larger tumor size (>15 cm: 9.5% and 32.5%; 10–15 cm: 13.2% vs. 22.5%, *p* < 0.001), younger age (59.1 ± 14.91 vs. 37.2 ± 19.05; *p* < 0.001), single status (17.0% vs. 45.0%; *p* < 0.001), single malignant tumor in a lifetime (76.1% vs. 87.5%; *p* = 0.0244), and pathologic grade IV diseases (14.5% vs. 40.0%; *p* < 0.001) compared with non-SCCO. After balancing the baseline clinical characteristics with a 1:4 ratio PSM, a total of matched 72 patients with SCCO and 254 patients with non-SCCO were identified. The survival rate of SCCO was distinctly inferior to non-SCCO, particularly in FIGO I, II, and III stages. Lymphadenectomy was performed in 37 (51.39%) SCCO patients, of whom 12 (32.43%) were found to have pathologically positive lymph nodes. Lymphadenectomy was linked to favorable overall survival in SCCO, particularly in the advanced stage, and was also an independent prognostic factor, whereas lymphadenectomy did not reveal an edge in matched non-SCCO. There was a pronounced survival benefit for SCCO when at least 10 or more nodes were resected. Lymphadenectomy in a non-stage-dependent way should be considered and deserves further clinical validation to promote the overall survival in SCCO.

## 1. Introduction

Small cell carcinoma of the ovary (SCCO) is a minority of extra-pulmonary small cell carcinoma (EPSCC) and consists of small cell carcinoma of the ovary-hypercalcemic type (SCCOHT) and small cell carcinoma of the ovary-pulmonary type (SCCOPT), accounting for less than 1% of all ovarian cancers [1]. Regardless of the diverse organs of origin, histopathological features of most EPSCC including SCCOPT tend to mirror those of the pulmonary small cell carcinoma with characteristics typical of small cell neuroendocrine carcinoma [1]. However, SCCOHT is an exception. With the presence of larger cells resembling malignant rhabdoid tumor [2], recurrent SMARCA4 mutations [3,4,5], SCCOHT is the unique subtype that does not belong to the family of neuroendocrine tumors but resembles malignant rhabdoid tumor.

Both types of SCCO are known for their poor prognosis. Only 33% of patients with stage IA SCCOHT have chances for long-term disease-free survival, and almost all the patients with tumors at a stage higher than IA died of disease [2]. The prognosis of SCCOPT is not much better either, with the median survival time less than 20 months even in FIGO stage I or II [6,7]. Salient differences in clinical characteristics between these two types include age, laterality, and hypercalcemia. Most patients with SCCOHT are young women, with a mean age of 24 at diagnosis compared to a mean age of 51 at diagnosis for SCCOPT. Patients with SCCOHT almost exclusively present with unilateral disease, while half of patients with SCCOPT present with bilateral disease. Approximately two thirds of patients with SCCOHT have hypercalcemia, which is almost absent in SCCOPT patients [2]. Additionally, SCCOPT has been found to frequently coexist with other gynecologic tract neoplasms [6,7].

Although both these subtypes are scarce, SCCOPT is even rarer, with only 38 cases reported in the literature before 2013 [6,8]. Given the rarity, heterogeneous management strategies are often administered in case reports or small retrospective series. Predictors of survival and optimal curative options [9,10], especially lymphadenectomy, have not been well delineated. Our study aimed at using the SEER database from 1975 to 2018 to discern latent prognostic factors that may inform clinical efforts and point the way to clinical trials.

## 2. Materials and Methods

Based on the database of SEER Research Plus Data, 9 Registries, Nov 2020 Sub (1975–2018), we extracted data for patients with primary site labeled ovary by SEER*Stat, version 8.4.0. Analyses were conducted with R, version 4.0.4. All cases of microscopically confirmed ovarian cancer and active follow-up were selected. The distribution of demographic, clinicopathological, and therapeutic characteristics was compared using chi-square tests. Student *t*-tests were used to assess the significance of differences in the mean values of continuous variables. Survival curves were estimated using the Kaplan–Meier method, with log-rank test to calculate statistical differences. Cox regression analyses were performed to evaluate the effects of variables on overall survival (OS). A 1:4 propensity score matching (PSM) analysis was employed to balance baseline variables for further analyses. A two-tailed *p*-value < 0.05 was considered statistically significant.

**Figure 1:**
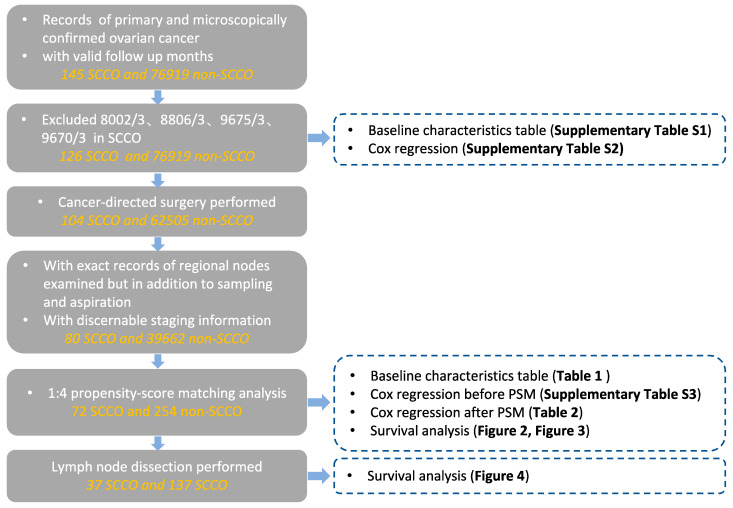
Flow chart of data selection.

For analysis purposes, two histology groups were created: small cell carcinoma of ovary consisting of ICD-O-3 Hist/behav code 8041/3 (small cell carcinoma, NOS) and 8044/3 (small cell carcinoma, intermediate cell), and non-small cell carcinoma of ovary. The codes 8002/3, 8806/3, 8045/3, 9675/3, and 9670/3 were ruled out for the SCCO group due to the inclusion of other malignancies characterized by small cells (e.g., malignant lymphoma). Data drawn from the “regional nodes examined/positive” were utilized to discriminate whether lymphadenectomy was performed. For analysis purposes, two lymph node dissection (LND) groups were formed: LND1 (1–10 lymph nodes removed) and LND2 (>10 lymph nodes removed). As described in the previous study [11], the log odds of positive lymph nodes (LODDS) were estimated by log (pnod + 0.5)/(tnod-pnod + 0.5), where pnod was the number of positive nodes and tnod was the total number of examined nodes. Usually, ovarian cancer in an early stage is defined as a stage lower than IIB, but some of the staging data in SEER are not precise enough for us to distinguish between stage IIA and stage IIB. To reduce the exclusion of SCCO data, the T1 or T2, and M0 (FIGO I-IIIA1), were defined as early stages, including those with a confined tumor but positive lymph node (FIGO IIIA1) and tumor extended to and/or implanted on other pelvic tissues (FIGO IIB). The rest were defined as advanced diseases. Between 1975 and 2018, a total of 126 patients with SCCO and 76,919 patients with non-SCCO through quality control and filter were identified. After eliminating records without cancer-directed surgery or with unspecific surgery information or no surgical procedure of primary site, excluding records without exact documentation of regional nodes examined or representing aspiration, sampling, and other unspecific information, we obtained 39,662 records on non-SCCO and 80 records on SSCO. The detailed selection procedure is summarized in Figure 1.

## 3. Results

### 3.1. Patients and Characteristics

Between 1975 and 2018, a total of 126 patients with SCCO through quality control and filter were identified. Baseline demographic, clinicopathologic, and therapeutic characteristics among patients with SCCO are summarized in Supplementary Table S1. The mean age of onset in SCCO patients was 43.0 years old. The majority of SCCO patients were white (80.2%), not single (60.3%), had only one malignancy in a lifetime (86.5%), and presented with unilateral disease (77.0%). The percentages of I, II, III, IV and Unknown/other FIGO stage were 22.2%, 8.7%, 23.0%, 30.2% and 15.9%, respectively. The percentage of diagnosis year before 1988 was relatively small (6.3%) and similar for each decade after 1988 (23.8%, 33.3%, 36.5% for 1988–1997, 1998–2007, 2008–2018, respectively). In terms of therapy, 74.6% of patients with SCCO received chemotherapy and 81.0% underwent surgery. Only 7.1% of patients received radiation therapy.

To identify risk factors for SCCO, univariable and multivariable cox regression were performed. Marital status, laterality, age, year of diagnosis, FIGO stage, chemotherapy, and surgery were bound up with better OS. After adjusting confounding factors, the independent prognosis factor consisted of chemotherapy (HR = 0.25, *p* < 0.001), surgery (HR = 0.31, *p* = 0.0012), and year of diagnosis (HR = 0.23, *p* = 0.0273; HR = 0.10, *p* < 0.001; HR = 0.17, *p* = 0.0055 for 1988–1997, 1998–2007 and 2008–2018, respectively, compared with 1975–1987) (Supplementary Table S2).

Like other pathological types of ovarian cancer, surgery and chemotherapy are key factors affecting the prognosis of SCCO. To further compare the differences in prognosis between SCCO and non-SCCO in terms of surgical modalities including lymph node dissection and clinicopathological features, we targeted patients who underwent cancer-directed surgery (deleting records with unspecific surgery information or no surgical procedure at the primary site) and had exact records of regional nodes examined (removing aspiration, sampling, and other unspecific records) and intact staging information in non-SCCO and SCCO. After filtering, we obtained 39,662 records on non-SCCO and 80 records on SSCO. Single status (17.0% vs. 45.0%, *p* < 0.001), single malignant tumor (76.1% vs. 87.5%, *p* = 0.0244), and pathologic grade IV diseases (14.5% vs. 40.0%, *p* < 0.001) were more common in SCCO patients. Patients with SCCO were prone to be unilateral tumor (57.6% and 85.0%, *p* < 0.001) and larger tumor size (>15 cm: 9.5% vs. 32.5%, 10–15 cm: 13.2% vs. 22.5%, *p* < 0.001). SCCO patients had a younger mean age of onset than non-SCCO patients (59.1 ± 14.91 vs. 37.2 ± 19.05, *p* < 0.001). Other characteristics, including race, year of diagnosis, FIGO stage, radiation therapy, chemotherapy, type of surgery, and lymphadenectomy, were parallel between SCCO and non-SCCO (Table 1). Given the confounding factors between groups of SCCO and non-SCCO, a 1:4 ratio PSM was employed to balance the baseline clinical characteristics. There were 72 cases of SCCO and 254 cases of non-SCCO finally matched. No variable achieved a significant difference between SCCO and none-SCCO after matching. Patients and characteristics before and after PSM were also exhibited in Table 1.

### 3.2. Survival and Prognostic Analysis

**Figure 2:**
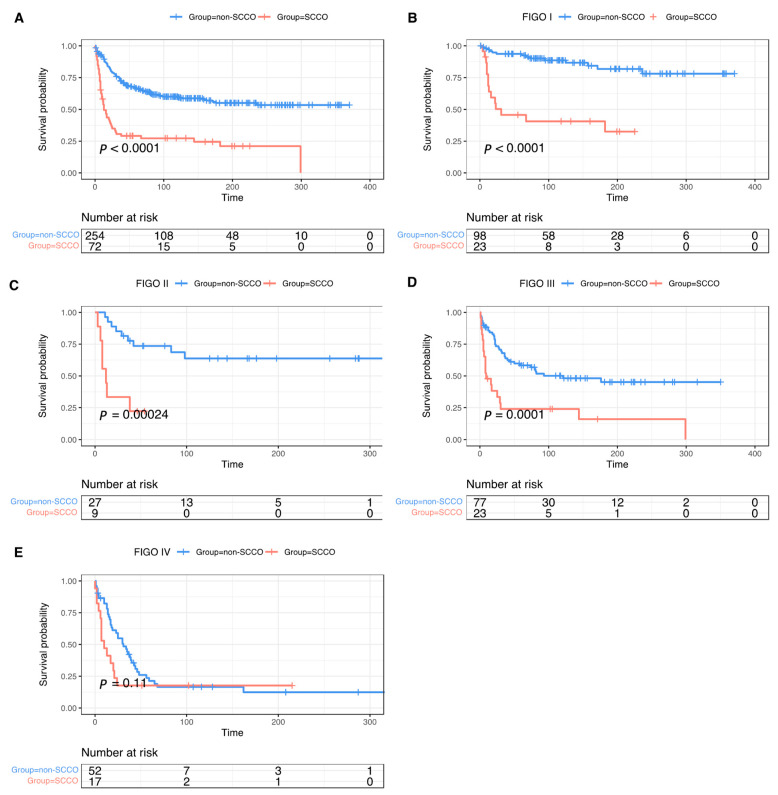
Survival curves of overall survival among patients with SCCO and non-SCCO. All stages (**A**), stage I (**B**), stage II (**C**), stage III (**D**), and stage IV (**E**) in matched cohort.

Patients with SCCO showed poorer survival than patients with non-SCCO (Log-rank *p* < 0.0001) (Figure 2A). When stratified by FIGO stage, SCCO presented less favorable outcomes in all stages except stage IV (Figure 2B–E). Univariable and multivariable cox regression were performed to assess risk factors for OS before (Supplementary Table S3) and after PSM (Table 2). In univariable cox regression analysis of the matched cohort, early FIGO stage, unilateral disease, and lymphadenectomy associated with better OS in SCCO and non-SCCO. However, unlike higher stages related to a higher risk of death in non-SCCO (HR = 2.61, *p* = 0.025; HR = 4.63, *p* < 0.001; HR = 11.23, *p* < 0.001 for stage II, stage III, and stage IV, respectively, compared with stage I), only advanced stage IV (HR = 2.2, *p* = 0.038) was a risk factor compared with stage I in SCCO. What remained statistically significant after multivariable cox regression analysis was unilateral disease, both in SCCO and non-SCCO. As for lymphadenectomy, it persistently benefited OS for SCCO (HR = 0.5, *p* = 0.0459), while for non-SCCO (HR = 0.82, *p* = 0.3903), it became insignificant after adjusting confounding factors. For SCCO (HR = 1.18, *p* = 0.7327 for stage IV compared with stage I), FIGO stage no longer predicted OS in multivariate analysis, but the late FIGO stage continued to be an essentially adverse independent prognostic factor for non-SCCO (HR = 3.4, *p* < 0.001; HR = 7.19, *p* < 0.001 for stage III and stage IV, respectively, compared with stage I). Furthermore, having more than one malignancy in a lifetime (HR = 2.73, *p* = 0.0427) and having a diagnosis between 1998 and 2007 (HR = 0.37, *p* = 0.0149) presented to be additional independent prognostic factors for SCCO. For non-SCCO, age older than 60 (HR = 3.67, *p* < 0.001 for ≥60 compared with <40) was another element compromising OS after adjusting confounding factors.

### 3.3. The Effect of Lymphadenectomy and Role of Lymph Node Metastasis

**Figure 3:**
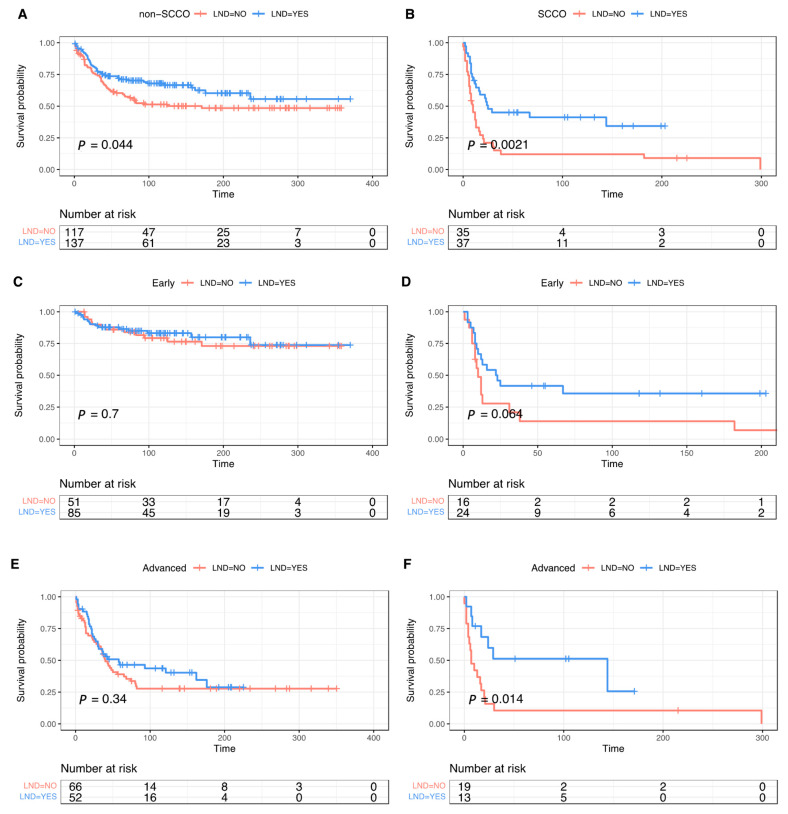
Survival curves of overall survival among patients with all stage (**A**,**B**), early stage (**C**,**D**), and advanced stage (**E**,**F**) in non-SCCO and SCCO in matched cohort divided by lymphadenectomy.

**Figure 4:**
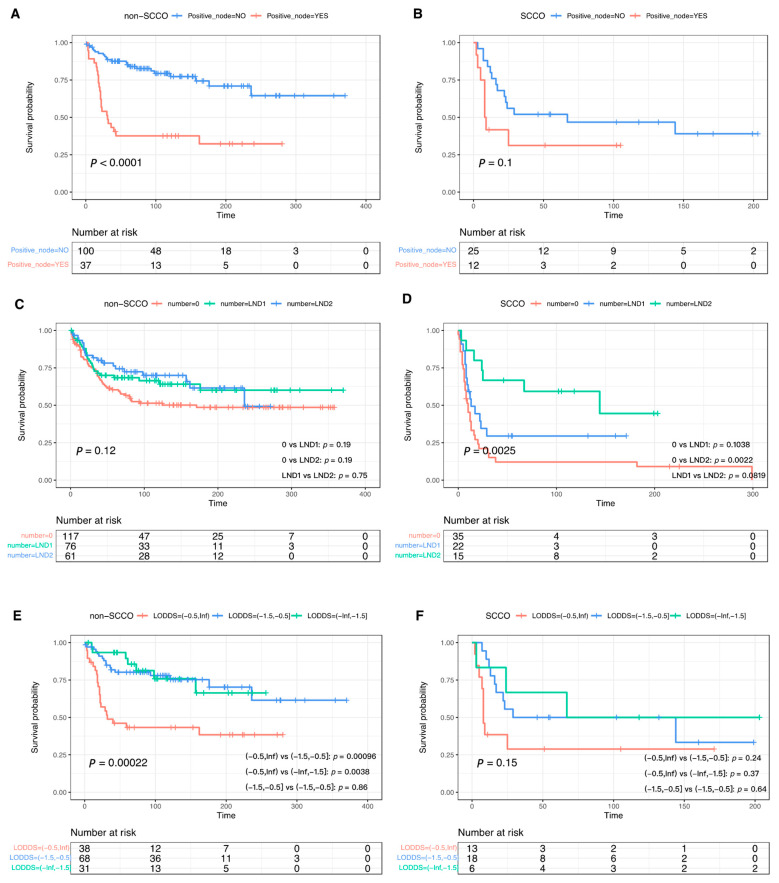
Overall survival curves for non-SCCO (**A**) and SCCO (**B**) patients who underwent lymphadenectomy stratified by positive lymph node. Overall survival curves among patients with non-SCCO (**C**) and SCCO (**D**) stratified by the number of resected lymph nodes. Overall survival curves among patients with non-SCCO (**E**) and SCCO (**F**) stratified by LODDS.

LND was performed in 37 (51.39%) SCCO patients, of whom 12 (32.43%) were found to have pathologically positive lymph nodes. In non-SCCO, there was 37 (27%) lymph nodes positive out of 137 (53.94%) individuals. Lymphadenectomy demonstrated significant overall survival benefit in non-SCCO (median OS: 171 months vs. not reached; Log-rank *p* = 0.044) (Figure 3A) and SCCO (median OS: 10 vs. 25 months; Log-rank *p* = 0.0021) (Figure 3B). There was a significant statistical difference in OS between non-LND and LND groups in the advanced stage (median OS: 7 vs. 144 months; Log-rank *p* = 0.014) (Figure 3F), but a marginal significance for apparently early stage (median OS: 10 months vs. 22.5 months; Log-rank *p* = 0.064) (Figure 3D) in SCCO. For non-SCCO, the survival benefit of lymphadenectomy faded when stratified according to early and late stages (Figure 3C,E). In cases undergoing lymphadenectomy, pathologically positive lymph nodes did not appear to impinge on the survival benefit of SCCO compared with the group without lymph node metastases but was a notable detriment for non-SCCO (*p* < 0.0001) (Figure 4A,B).

As LND theoretically removes positive lymph nodes to improve prognosis, we further investigate whether LND and LOODS brought ultimate survival benefit. There was a tendency that the more lymph nodes removed, the better OS in SCCO, but not in non-SCCO. For non-SCCO patients, regardless of the number of lymph nodes, lymphadenectomy was not conducive (*p* = 0.12) (Figure 4C). Patients in the LND2 group had a superior survival rate than patients without lymphadenectomy (adjusted Log-rank *p* = 0.0022), whereas the survival benefit was not achieved in patients with 1 to 10 lymph nodes removed (Figure 4D). LODDS was not amenable to predicting prognosis in SCCO (Log-rank *p* = 0.15) (Figure 4F), but the opposite was true for non-SCCO (Figure 4E).

## 4. Discussion

Over the past decades, the optimal curative planning, including lymphadenectomy for SCCO, has remained uncharted, given the highly individualized therapeutic pattern and lack of consensus in cancer management. In addition, few studies have retrospectively examined SCCO from the perspective of treatment strategies. Using the SEER database and grounded in curative strategies, we retrospectively investigated features that shape overall survival in SCCO. Consistent with previous findings, the survival rate of SCCO was notably worse compared to non-SCCO. Lymphadenectomy was associated with favorable overall survival in SCCO, especially in the advanced stage. Resection of at least 10 nodes may exert a striking survival influence for SCCO.

In non-SCCO, chemotherapy and lymphadenectomy reduced the risk of death before PSM, but this advantage subsided after balancing the baseline difference between SCCO and non-SCCO, which indicated baseline characteristics, especially marital status, number of malignancies in a lifetime, grade, laterality, age, and tumor size, are likely to be confounding factors for impaired survival in chemotherapy and lymphadenectomy. Notably, lymphadenectomy was an independent prognostic factor for SCCO before and after PSM in our study. LND, particularly in the advanced stage or with more than 10 nodes removed, was correlated with favorable OS. Even though lymph node dissection was associated with better OS, positive lymph node status or LODDS were not prognostic factors to evaluate the invasiveness and progression of the disease as described in other types of ovarian cancer [11,12]. The results support a non-stage-dependent lymphadenectomy strategy in SCCO, which is in accordance with the management of adolescents and young adults with SCCOHT in ESGO–SIOPE guideline. In the advanced stage, it recommends full pelvic and para-aortic lymphadenectomy if complete removal of peritoneal disease can be achieved [13]. Nevertheless, the strategy of systematic lymphadenectomy is somewhat different in the most common epithelial ovarian cancer and has long been an area of controversy due to the ambiguous conclusions on whether lymphadenectomy can translate into progress-free survival (PFS) or OS promotion [14]. Although lymphadenectomy could theoretically remove and increase detection of the potentially metastatic lesion, the benefit should be weighed against complications, including blood loss, longer operating times, and hospital stays. Based on the result of a large, randomized trial (LION, NCT00712218) [15], in newly diagnosed invasive epithelial ovarian cancer involving the pelvis and upper abdomen (stage ≥ IIB), resection of clinically negative nodes is not required in NCCN guidelines [16]. Although a retrospective study including 469 cases of SCCO between 2004 and 2014 concluded that the performance of LND was not associated with better OS, this conclusion was not stratified by the number of lymph nodes resected or by FIGO staging, so some potentially positive findings may have been omitted [17].

Although chemotherapy reduced the risk of death by 46% in SCCO, chemotherapy was not an independent predictor of survival in SCCO either before or after PSM in our study. On the one hand, it illustrated the highly aggressive nature of SCCO. On the other hand, most patients in our cohort may not have received the optimal chemotherapy regimen. Early on, the choice of regimen for SCCO was generally extrapolated from data in small cell lung carcinoma that was full of heterogeneity. With the publication of two prospective studies in SCCOHT, it was after 2018 that guidelines recommended a high-dose chemotherapy regimen (HDC) for patients who achieved a complete response (CR) after optimal cytoreductive surgery and PAVEP for four to six cycles with autologous stem cell transplantation (ASCT) rescue [13,18]. In the retrospective research, chemotherapy regimens for SCCOPT mainly consist of carboplatin or cisplatin, etoposide, and to a lesser extent alkylating agents, paclitaxel, and irinotecan, and there was a trend towards improved survival with the use of etoposide and anthracyclines [6]. However, the chemotherapy regimen for SCCOPT has not yet reached a consensus so far due to lack of prospective studies. Therefore, with the widespread use of optimal chemotherapy regimens, the role of chemotherapy in prolonging survival in SSCO will become more prominent in future retrospective studies.

Similar to the finding that patients who received HDC followed by pelvic radiotherapy did not exhibit significantly better outcomes compared to those who did not receive irradiation in prospective research [19], radiation was not an independent predictor of survival in SCCO, either before or after PSM. However, there are reports confirming prolonged responses and improved survival rates with adjuvant radiotherapy in SCCOHT [17,20,21]. There are also reports of patients with stage IIIC SCCOPT who had been disease-free for more than four years after completing adjuvant therapy and receiving consolidation radiotherapy [22]. The value of radiotherapy in the treatment of SCCO remains to be proven.

We studied rare tumors by accessing data from public databases and uncovered unrecognized features of rare tumor. To our knowledge, our study is currently the only one that supports a non-stage-dependent lymphadenectomy strategy for SCCO. This strategy is a departure from the most common epithelial ovarian cancer and warrants further study because of the potential to guide surgical strategy and improve survival. Nevertheless, despite these strengths, several limitations of our study should be noted. Of the 126 cases of SCCOs included, only two had a pathological type of 8044/3: small cell carcinoma, intermediate cell (the 2020 WHO classification was SCCOHT), and the remaining was 8041/3: small cell carcinoma, NOS (the 2020 WHO classification was neuroendocrine carcinoma of ovary, and it was termed as SCCOPT before [23]), which was inconsistent with previous literature reporting that the incidence of SCCOPT was much lower than that of SCCOHT. This may be attributable to the misclassification. Somatic or germline mutation in SMARCA4 were not identified as an essential molecular feature of SCCOHT until the last decade. Correspondingly, one of the diagnostic hallmarks of SCCOHT, the loss of SMARCA4/BRG1 in immunohistochemistry, has only been widely acknowledged in recent years [24]. Our study reviewed over 30 years of data from SSER. Thus, SCCOHT may have been previously misclassified due to the lack of characteristic diagnostic markers. It was for this reason that we were unable to discriminate between the hypercalcemic and pulmonary subtypes of SCCO, and we analyzed these two types as a whole. As mentioned above, the two types have divergent molecular, clinical, and pathological features. Thus, conflating them together somewhat undermined accuracy. Furthermore, detailed information on chemotherapy regimens and the extent of surgery were not available from SEER, restricting the inquiry into some vital variables.

Our research backs up the strategy of a non-stage-dependent lymphadenectomy. However, it is a meager effort to overcome the therapeutic challenges of this rare tumor. With the ongoing knowledge, more accurate pathological diagnosis will help us study the two different subtypes of small cell carcinoma in greater depth in future retrospective studies. Moreover, more extensive multi-center collaborations should be established to complete higher-quality clinical trials on SCCO, where the lymphadenectomy strategy mentioned above should be considered and validated. In addition, the management of SCCO warrants treatments with valid preclinical evidence. Epigenetic therapeutics [25], kinase inhibitors [26], and immunotherapies [27] should be considered for addition to current multimodal therapy or as post-relapse therapy. Lastly, based on the unique molecular characteristics of SCCOHT, the development of more effective targeted drugs cannot be disregarded.

## Figures and Tables

**Table 1:** Baseline demographic, clinicopathologic, and therapeutic characteristics for SCCO and non-SCCO before and after propensity matching.

Clinical Parameter	Unmatched Dataset			Matched Dataset (4:1)		
	Non-SCCO	SCCO	*p*-Value	Non-SCCO	SCCO	*p*-Value
	(*n* = 39,662)	(*n* = 80)		(*n* = 254)	(*n* = 72)	
Marital status						
Married and other	31,702 (79.9%)	43 (53.8%)	<0.001	148 (58.3%)	40 (55.6%)	0.85
Single	6762 (17.0%)	36 (45.0%)		101 (39.8%)	31 (43.1%)	
Unknown	1198 (3.0%)	1 (1.3%)		5 (2.0%)	1 (1.4%)	
Race						
Black	2564 (6.5%)	7 (8.8%)	0.468	26 (10.2%)	7 (9.7%)	0.89
Other	3430 (8.6%)	10 (12.5%)		37 (14.6%)	9 (12.5%)	
Unknown	100 (0.3%)	0 (0%)				
White	33,568 (84.6%)	63 (78.8%)		191 (75.2%)	56 (77.8%)	
Malignancy						
≥2	9463 (23.9%)	10 (12.5%)	0.0244	37 (14.6%)	10 (13.9%)	1
1	30,199 (76.1%)	70 (87.5%)		217 (85.4%)	62 (86.1%)	
Grade						
I	3464 (8.7%)	0 (0%)	<0.001			
II	6436 (16.2%)	0 (0%)				
III	13,787 (34.8%)	20 (25.0%)		77 (30.3%)	20 (27.8%)	0.9
IV	5758 (14.5%)	32 (40.0%)		79 (31.1%)	24 (33.3%)	
Unknown	10,217 (25.8%)	28 (35.0%)		98 (38.6%)	28 (38.9%)	
Laterality						
Bilateral	16,829 (42.4%)	12 (15.0%)	<0.001	50 (19.7%)	12 (16.7%)	0.685
Unilateral	22,833 (57.6%)	68 (85.0%)		204 (80.3%)	60 (83.3%)	
Age						
Mean (SD)	59.1 (14.9)	37.2 (19.1)	<0.001	40.9 (20.3)	39.4 (18.7)	0.548
Median [Min, Max]	60.0 [0, 100]	32.0 [10.0, 91.0]		41.0 [2.00, 89.0]	35.0 [14.0, 91.0]	
Year of diagnosis						
1988–1997	12,984 (32.7%)	22 (27.5%)	0.601	64 (25.2%)	17 (23.6%)	0.956
1998–2007	13,608 (34.3%)	29 (36.3%)		95 (37.4%)	28 (38.9%)	
2008–2018	13,070 (33.0%)	29 (36.3%)		95 (37.4%)	27 (37.5%)	
size						
>15 cm	3749 (9.5%)	26 (32.5%)	<0.001	65 (25.6%)	23 (31.9%)	0.809
10–15 cm	5224 (13.2%)	18 (22.5%)		51 (20.1%)	15 (20.8%)	
5–10 cm	7225 (18.2%)	10 (12.5%)		42 (16.5%)	10 (13.9%)	
0–5 cm	5946 (15.0%)	4 (5.0%)		20 (7.9%)	4 (5.6%)	
No/Micro	165 (0.4%)	0 (0%)				
Unknown	17,353 (43.8%)	22 (27.5%)		76 (29.9%)	20 (27.8%)	
FIGO stage						
I	11,647 (29.4%)	27 (33.8%)	0.475	98 (38.6%)	23 (31.9%)	0.764
II	3892 (9.8%)	10 (12.5%)		27 (10.6%)	9 (12.5%)	
III	15,088 (38.0%)	24 (30.0%)		77 (30.3%)	23 (31.9%)	
IV	9035 (22.8%)	19 (23.8%)		52 (20.5%)	17 (23.6%)	
Radiation						
No	38,759 (97.7%)	77 (96.3%)	0.612	249 (98.0%)	70 (97.2%)	1
Yes	903 (2.3%)	3 (3.8%)		5 (2.0%)	2 (2.8%)	
Chemotherapy						
No/Unknown	11,819 (29.8%)	18 (22.5%)	0.192	62 (24.4%)	17 (23.6%)	1
Yes	27,843 (70.2%)	62 (77.5%)		192 (75.6%)	55 (76.4%)	
Surgery type						
DEB/EXE	3982 (10.0%)	5 (6.3%)	0.463	16 (6.3%)	4 (5.6%)	0.956
Non-DEB	9002 (22.7%)	17 (21.3%)		48 (18.9%)	13 (18.1%)	
Unknown	26,678 (67.3%)	58 (72.5%)		190 (74.8%)	55 (76.4%)	
LND						
No	20,746 (52.3%)	39 (48.8%)	0.6	117 (46.1%)	35 (48.6%)	0.804
Yes	18,916 (47.7%)	41 (51.3%)		137 (53.9%)	37 (51.4%)	

No/Micro: no mass; no tumor found/microscopic focus or foci only; DEB/EXE: debulking surgery/pelvic exenteration; Non-DEB: non-debulking surgery; LND: lymph node dissection.

**Table 2:** Univariable and multivariable Cox regression models for overall survival among patients with non-SCCO and SCCO in matched cohort.

Characteristics		Non-SCCO				SCCO			
		Crude HR (95% CI)	*p*-Value	Adjusted HR (95% CI)	*p*-Value	Crude HR (95% CI)	*p*-Value	Adjusted HR (95% CI)	*p*-Value
Marital status									
Married and other		1				1			
Single		0.39 (0.24–0.62)	<0.001	0.89 (0.49–1.63)	0.7095	1.17 (0.68–2.03)	0.566		
Unknown		1.49 (0.54–4.08)	0.439	0.97 (0.33–2.9)	0.9629	6.08 (0.78–47.63)	0.086		
Race									
Black		1				1			
Other		0.49 (0.23–1.05)	0.066			0.67 (0.19–2.34)	0.536		
White		0.62 (0.35–1.07)	0.087			1.12 (0.44–2.84)	0.809		
Malignancy									
≥2									
1		0.74 (0.45–1.24)	0.251			2.6 (1.03–6.59)	0.044	2.73 (1.03–7.2)	0.0427
Grade									
III		1				1			
IV		1.14 (0.72–1.79)	0.572	1.31 (0.78–2.22)	0.3062	1.03 (1.03–6.59)	0.926		
Unknown		0.55 (0.33–0.91)	0.02	0.93 (0.52–1.64)	0.7908	1.03 (0.52–2.03)	0.941		
Laterality									
Bilateral		1				1			
Unilateral		0.25 (0.16–0.37)	<0.001	0.42 (0.27–0.67)	<0.001	0.33 (0.17–0.63)	0.001	0.43 (0.19–0.95)	0.0365
Age									
<40		1				1			
40–59		3.33 (1.98–5.62)	<0.001	1.62 (0.77–3.42)	0.2025	0.54 (0.17–0.63)	0.078		
≥60		7.6 (4.33–13.33)	<0.001	3.67 (1.7–7.93)	<0.001	1.06 (0.47–2.38)	0.892		
Year of diagnosis									
1988–1997		1				1			
1998–2007		0.8 (0.5–1.26)	0.337			0.33 (0.16–0.65)	0.001	0.37 (0.17–0.82)	0.0149
2008–2018		0.68 (0.4–1.13)	0.139			0.53 (0.27–1.05)	0.069	0.91 (0.39–2.16)	0.8356
Size									
0–5 cm		1				1			
5–10 cm		0.51 (0.25–1.02)	0.058	0.63 (0.3–1.31)	0.2202	2.46 (0.51–11.96)	0.263		
10–15 cm		0.36 (0.18–0.74)	0.005	0.55 (0.26–1.2)	0.136	2.98 (0.66–13.5)	0.156		
>15 cm		0.34 (0.17–0.68)	0.002	0.82 (0.38–1.81)	0.6302	1.56 (0.35–6.9)	0.559		
Unknown		0.53 (0.28–1)	0.049	0.55 (0.28–1.07)	0.0786	3.01 (0.69–13.22)	0.144		
FIGO stage									
I		1							
II		2.61 (1.13–6.04)	0.025	2.11 (0.88–5.04)	0.0924	1.86 (0.75–4.66)	0.183	1.65 (0.65–4.19)	0.2922
III		4.63 (2.5–8.55)	<0.001	3.4 (1.77–6.53)	<0.001	1.94 (0.96–3.91)	0.065	1.55 (0.73–3.28)	0.2561
IV		11.23 (6.05–20.84)	<0.001	7.19 (3.52–14.7)	<0.001	2.2 (0.96–3.91)	0.038	1.18 (0.46–2.99)	0.7327
Radiation									
No		1							
Yes		0.68 (0.09–4.88)	0.7			0.65 (0.09–4.74)	0.675		
Chemotherapy									
No/Unknown		1				1			
Yes		1.05 (0.67–1.65)	0.837			0.54 (0.3–0.96)	0.037	0.54 (0.28–1.04)	0.0639
Surgery									
DEB/EXE		1				1			
Non-DEB		0.47 (0.23–0.96)	0.039	2.53 (1.19–5.38)	0.0162	0.99 (0.32–3.08)	0.983		
Unknown		0.44 (0.24–0.81)	0.008	1.7 (0.81–3.6)	0.1627	0.4 (0.32–3.08)	0.088		
LND									
No		1				1			
Yes		0.67 (0.45–0.99)	0.045	0.82 (0.53–1.28)	0.3903	0.43 (0.32–3.08)	0.003	0.5 (0.25–0.99)	0.0459

No/Micro: no mass; no tumor found/microscopic focus or foci only; DEB/EXE: debulking surgery/pelvic exenteration; Non-DEB: non-debulking surgery; LND: lymph node dissection.

## Data Availability

The datasets analyzed in this study can be found in the SEER website of the National Cancer Institute (http://seer.cancer.gov (accessed on 20 March 2022)).

## Supplementary Materials

The following supporting information can be downloaded at: https://www.mdpi.com/article/10.3390/curroncol29100617/s1, Table S1: Baseline demographic, clinicopathologic and therapeutic characteristics for SCCO, Table S2: Cox regression models for overall survival among 126 cases of small cell carcinoma of ovary, Table S3: Univariable and multivariable Cox regression models for overall survival among patients with non-SCCO and SCCO in unmatched cohort.
