# Supplementary material for: Lymphadenectomy Benefits Small Cell Carcinoma of Ovary: A Population-Based Analysis
Source: Curr Oncol. 2022 Oct 16;29(10):7802–15. doi: 10.3390/curroncol29100617 (PMC9600050; doi:10.3390/curroncol29100617)
Supplement: Supplementary file 1 [file curroncol-29-00617-s001.zip › curroncol-1900381-supplementary.pdf]

Supplementary Table S1. Baseline demographic, clinicopathologic and therapeutic characteristics for SCCO.

	**Small (N=126)**
**Marital status**	
Married and other	73 (57.9%)
Single	50 (39.7%)
Unknown	3 (2.4%)
**Race**	
Black	10 (7.9%)
Other	14 (11.1%)
Unknown	1 (0.8%)
White	101 (80.2%)
**Malignance**	
≥2	17 (13.5%)
1	109 (86.5%)
**Grade**	
I	0 (0%)
II	1 (0.8%)
III	25 (19.8%)
IV	48 (38.1%)
Other	0 (0%)
Unknown	52 (41.3%)
**Laterality**	
Bilateral	29 (23.0%)
Unilateral	97 (77.0%)
**Age**	
Mean (SD)	43.0 (21.4)
Median [Min, Max]	38.0 [10.0, 91.0]
**Year of diagnosis**	
1975-1987	8 (6.3%)
1988-1997	30 (23.8%)
1998-2007	42 (33.3%)
2008-2018	46 (36.5%)
**size**	
>15cm	28 (22.2%)
10-15cm	22 (17.5%)
5-10cm	13 (10.3%)
0-5cm	6 (4.8%)
No/Micro	0 (0%)
Unknown	57 (45.2%)
**FIGO**	
I	28 (22.2%)
II	11 (8.7%)

III	29 (23.0%)
IV	38 (30.2%)
Unknown/other	20 (15.9%)
**Radiation**	
No	117 (92.9%)
Yes	9 (7.1%)
**Chemotherapy**	
No/Unknown	32 (25.4%)
Yes	94 (74.6%)
**Surgery**	
No	23 (18.3%)
Yes	102 (81.0%)
Unknown/other	1 (0.8%)

No/Micro: no mass; no tumor found/microscopic focus or foci only

Supplementary Table S2. Cox regression models for overall survival among 126 cases of small cell carcinoma of ovary.

Covariate, level	Crude HR (95% CI)	p-value	Adjusted HR (95% CI)	p-value
**Marital status**				
Married and other	1			
Single	0.84(0.55 - 1.28)	0.425	1.23(0.74 - 2.03)	0.4257
Unknown	4.82(1.46 - 15.92)	0.01	2.76(0.68 - 11.19)	0.155
**Race**				
Black	1			
White	0.95(0.46 - 1.96)	0.879		
Other	0.64(0.24 - 1.7)	0.366		
Unknown	0(0 - Inf)	0.996		
**Malignance**				
≥2	1			
1	1.72(0.89 - 3.32)	0.108		
**Grade**				
III	1			
IV	1.25(0.71 - 2.22)	0.445		
Unknown	1.26(0.71 - 2.23)	0.426		
II	0(0 - Inf)	0.995		
**Laterality**				
Bilateral	1			
Unilateral	0.3(0.19 - 0.47)	<0.001	0.64(0.36 - 1.15)	0.1332
**Age**				
<40	1		1	
40-59	0.82(0.48 - 1.41)	0.47	0.56(0.3 - 1.06)	0.0752
≥60 years	2.75(1.71 - 4.43)	<0.001	1.55(0.3 - 1.06)	0.2549
**Year of diagnosis**				
1975-1997	1			

1988-1997	0.42(0.19 - 0.94)	0.035	0.23(0.06 - 0.85)	0.0273
1998-2007	0.21(0.09 - 0.48)	<0.001	0.10(0.06 - 0.85)	<0.001
2008-2018	0.27(0.12 - 0.59)	0.001	0.17(0.05 - 0.59)	0.0055
**Size**				
0-5cm	1			
5-10cm	1.92(0.6 - 6.14)	0.271		
10-15cm	2.05(0.69 - 6.03)	0.194		
> 15cm	0.86(0.29 - 2.58)	0.792		
Unknown	1.78(0.64 - 4.97)	0.27		
**FIGO stage**				
I	1			
II	1.93(0.86 - 4.36)	0.112	1.25(0.55 - 2.88)	0.5941
III	1.86(0.98 - 3.51)	0.056	1.66(0.85 - 3.27)	0.1395
IV	2.89(1.6 - 5.22)	<0.001	1.7(0.78 - 3.71)	0.1835
**Radiation**				
No	1			
Yes	0.69(0.28 - 1.7)	0.419		
**Chemotherapy**				
No/Unknown	1			
Yes	0.36(0.23 - 0.56)	<0.001	0.25(0.15 - 0.43)	<0.001
**Surgery**				
No	1			
Yes	0.27(0.16 - 0.44)	<0.001	0.31(0.16 - 0.63)	0.0012
Unknown/other	6.36(0.81 - 50.17)	0.079	8.7(0.87 - 87.14)	0.0658

Supplementary Table S3. Univariable and multivariable Cox regression models for overall survival among patients with non-SCCO and SCCO in unmatched cohort.

Characteristics	non-SCCO				SCCO			
	Crude HR (95% CI)	p-value	Adjusted HR (95% CI)	p-value	Crude HR (95% CI)	p-value	Adjusted HR (95% CI)	p-value
**Marital status**								
Married and other	1				1			
Single	0.64	<0.001	1.02(0.98 - 1.06)	0.3328	1.06(0.63 - 1.77)	0.835		
Unknown	0.82	<0.001	0.93(0.86 - 1)	0.0637	5.94(0.77 - 46.16)	0.088		
**Race**								
Black	1				1			
Other	0.69	<0.001	0.79(0.74 - 0.85)	<0.001	0.77(0.23 - 2.53)	0.664		
Unknown	0.18	<0.001	0.33(0.19 - 0.56)	<0.001				
White	0.96	0.117	0.85(0.81 - 0.89)	<0.001	1.11(0.44 - 2.79)	0.829		
**Malignance**								
≥2	1				1			
1	1.16	<0.001	1.13(1.1 - 1.16)	<0.001	2.57(1.02 - 6.48)	0.045	2.79(1.06 - 7.33)	0.0373
**Grade**								
1	1							
2	1.88(1.76 - 2)	<0.001	1.34(1.26 - 1.43)	<0.001				
3	3.33(3.15 - 3.53)	<0.001	1.57(1.48 - 1.66)	<0.001	1			
4	3.05(2.86 - 3.24)	<0.001	1.53(1.43 - 1.63)	<0.001	1.03(0.54 - 1.99)	0.927		
Unknown	2.11(1.99 - 2.24)	<0.001	1.39(1.31 - 1.48)	<0.001	1.03(0.54 - 1.99)	0.927		
**Laterality**								
Bilateral	1				1			
Unilateral	0.46	<0.001	0.84(0.82 - 0.86)	<0.001	0.34(0.18 - 0.65)	0.001	0.37(0.16 - 0.86)	0.0211
**Age**								
<40	1				1			
≥60	6.06(5.67 - 6.48)	<0.001	3.79(3.53 - 4.07)	<0.001	1.07(0.48 - 2.38)	0.878		
40-59	2.94(2.75 - 3.15)	<0.001	2.24(2.09 - 2.41)	<0.001	0.55(0.28 - 1.08)	0.081		
**Year of diagnosis**								
1988-1997	1				1			
1998-2007	0.86	<0.001	0.89(0.86 - 0.93)	<0.001	Year21998-2008	0.3(0.16 - 0.58)	0.59(0.18 - 1.95)	0.3908
2008-2018	0.71	<0.001	0.79(0.75 - 0.82)	<0.001	Year22008-2018	0.49(0.26 - 0.92)	1.38(0.39 - 4.92)	0.617
**Size**								
0-5cm	1				1			
5-10cm	1.07	0.004	0.97(0.93 - 1.01)	0.1724	2.47(0.51 - 12)	0.261		
10-15cm	0.88	<0.001	0.95(0.9 - 1)	0.0361	3.16(0.71 - 14.01)	0.131		

>15cm	0.75	<0.001	0.99(0.93 - 1.05)	0.7053	1.43(0.32 - 6.28)	0.638		
Unknown	1.37	<0.001	1.08(1.04 - 1.12)	0.0001	3.27(0.75 - 14.24)	0.115		
No/Micro	0.39	<0.001	0.89(0.65 - 1.22)	0.4842				
**FIGO stage**								
I	1				1			
II	2.08(1.97 - 2.19)	<0.001	1.87(1.77 - 1.97)	<0.001	1.77(0.76 - 4.13)	0.187	1.21(0.51 - 2.88)	0.6632
III	4.5(4.34 - 4.67)	<0.001	3.53(3.38 - 3.69)	<0.001	1.68(0.86 - 3.27)	0.126	1.35(0.66 - 2.76)	0.406
IV	6.73(6.47 - 7)	<0.001	4.64(4.43 - 4.86)	<0.001	2.26(1.13 - 4.49)	0.02	1.24(0.55 - 2.8)	0.6095
**Radiation**								
No	1							
Yes	0.93	0.094			0.34(0.05 - 2.49)	0.291		
**Chemotherapy**								
No	1				1			
Yes	1.46(1.42 - 1.5)	<0.001	0.77(0.74 - 0.79)	<0.001	0.56(0.32 - 0.98)	0.042	0.65(0.34 - 1.27)	0.2088
**Surgery**								
DEB/EXE	1				1			
Non-DEB	0.49	<0.001	0.93(0.9 - 0.97)	0.0013	0.78(0.29 - 2.14)	0.632	1.67(0.51 - 5.52)	0.3973
Unknown	0.48	<0.001	NA		0.31(0.12 - 0.81)	0.017	NA	
**LND**								
No	1				1			
Yes	0.54	<0.001	0.7(0.69 - 0.72)	<0.001	0.42(0.25 - 0.71)	0.001	0.49(0.25 - 0.97)	0.0403

No/Micro: no mass; no tumor found/microscopic focus or foci only; DEB/EXE: debulking surgery/pelvic exenteration; Non-DEB: non-debulking surgery; LND: lymph node dissection.
